# Is EQ-5D-5L sensitive enough to detect treatment-related changes in health status of prostate cancer patients? A nationwide Norwegian longitudinal study from the prostate cancer registry

**DOI:** 10.1007/s11136-026-04178-z

**Published:** 2026-02-04

**Authors:** Marjolein M. Iversen, J. Kirkeleit, T. M. Norekvål, K. Oterhals, Y. Gjelsvik, T. B. Johannesen, K. Breivik

**Affiliations:** 1 Centre on Patient-Reported Outcomes, Department of Research and Development, Haukeland University Hospital, Bergen, Norway; 2 Department of Health and Caring Sciences, Western Norway University of Applied Sciences, Bergen, Norway; 3 Department of Occupational Medicine and Epidemiology, National Institute of Occupational Health, Oslo, Norway; 4 Department of Clinical Science, University of Bergen, Bergen, Norway; 5 Department of Heart Disease, Haukeland University Hospital, Bergen, Norway; 6 Cancer Registry of Norway, Norwegian Institute of Public Health, Oslo, Norway; 7 Regional Centre for Child and Youth Mental Health and Child Welfare, NORCE Norwegian Research Centre, Bergen, Norway

**Keywords:** Prostate Cancer, Patient-Reported Outcomes, Prospective Study, Sensitivity, Responsiveness to Change, Registry study

## Abstract

**Purpose:**

To examine the generic European Quality of Life 5-Dimensions questionnaire (EQ-5D-5L) responsiveness to change in health status of patients with invasive prostate cancer (PCa) from pre- to post-treatment and compare these results with the responsiveness of disease-specific instruments assumed to be particularly sensitive to change in a large sample of PCa patients.

**Methods:**

In this nationwide registry study (2017–2019), PCa patients in Norway (n = 620) and a matched reference group from the general population without previous cancer history (n = 1776) were invited. All participants completed the EQ-5D-5L, the European Organization for Research and Treatment of Cancer Quality of Life Questionnaire (EORTC-QLQ-C30) and the Expanded Prostate Cancer Index Composite short form (EPIC-26) pre- and post-treatment. Paired t-tests were performed with latent change scores in a structural equation model framework.

**Results:**

EQ-5D-5L showed minor changes from pre- to post-treatment for four dimensions and sum score (Cohen’s d ≤ 0.20). EORTC-QLQ-C30 revealed a moderate decline in social function (-9.85, p < 0.001, Cohen’s d -0.52). EPIC-26 showed a large decline in sexual function (-38.1, p < 0.001, Cohen’s d -1.42), and urine incontinency (-24.3, p < 0.001, Cohen’s d -1.86). Correlations between change in EQ-5D-5L and change in domains found to be important for participants with PCa (sexual function, urine incontinence and social function) revealed small to medium correlations for the PCa group.

**Conclusions:**

The low responsiveness indicates that the EQ-5D-5L is not sensitive enough in detecting health status changes due to significant problem areas in PCa patients. Adding disease-specific measures is pivotal to measuring these changes in health status.

**Supplementary Information:**

The online version contains supplementary material available at 10.1007/s11136-026-04178-z.

## Introduction

Prostate cancer (PCa) is the second most common cancer among men globally and the most prevalent among Norwegian men aged 50 years and above, accounting for 26.3% of all male cancer cases from 2019 to 2023 [1]. Survival rates have improved with prostate-specific antigen (PSA) testing, early detection, and treatment advances [2]. Surgery, radiotherapy and chemotherapy however are associated with both acute and long-term morbidity [3] and can substantially reduce health-related quality of life (HRQoL) [3, 4]. Consequently, accurate measurement of HRQoL using patient-reported outcomes measures (PROMs) is essential and their responsiveness, or sensitivity to clinically meaningful change, is a key requirement. In PCa patients, the responsiveness of a PROM such as the generic European Quality of Life 5-Dimensions questionnaire (EQ-5D-5L), depends both on its psychometric properties (including construct relevance, reliability, and sensitivity to change) and on the clinical context, such as disease status, treatment, and prognosis [5–7].

In real-world PCa populations, it remains uncertain whether EQ-5D-5L can adequately capture treatment-related changes in health status. We hypothesised that the EQ-5D-5L would demonstrate lower responsiveness compared with disease-specific PROMs.

The EQ-5D-5L is a generic, preference based instrument of health utility and health status. Its use in PCa may be limited by structural coverage gaps, such as the absence of sexual and urinary domains, as well as by measurement limitations, including ceiling effects that restrict its ability to detect change [5–10]. Although the EQ-5D-5L is often used to measure HRQoL in tandem with cancer-specific PROMs [11–13], it is also used as a stand-alone instrument to measure HRQoL [14], yet its capacity to reflect post-treatment changes in PCa remains uncertain [6].

The present study therefore examined the responsiveness of the EQ-5D-5L to changes in health status in a large cohort of PCa patients over a period of time when such change was expected (before /after treatment). EQ-5D-5L’s responsiveness to change was compared with similar analyses done on the cancer-specific European Organization for Research and Treatment of Cancer Quality of Life Questionnaire (QLQ-C30) and the Expanded Prostate Cancer Index Composite short form (EPIC-26), which are assumed to be particularly sensitive to change in this patient group.

## Methods

### Data sources

All incident cancer cases and pre-cancerous lesions are registered with the Cancer Registry of Norway (CRN) which includes information on basic diagnosis (location and morphology) and treatment-related data [14, 15]. Pathology reports are submitted directly from the pathology laboratories to the CRN, and the database is continuously updated and matched with information from The National Population Register on vital status and migration. The registration is compulsory, and it has been estimated that the CRN is more than 99% complete on a minimum of diagnostic information on solid tumors [16]. The Norwegian Prostate Cancer Registry (NoPCR) includes all incident PCa cases (ICD 10, localization C61) with individual information on pathological and clinical data sent by an electronic cancer report from the clinicians.

### Study population

In this nationwide prospective study (2017–2019), CRN invited all PCa patients diagnosed with invasive PCa in Norway from January 2017 through December 2019 to participate in a three-year survey on Men’s Health. The survey collected patient-reported outcomes (PROs) on a large scale with the intention to test and justify whether PROMs should be implemented for all new incident PCa cases in the Norwegian Prostate Cancer Registry (NoPCR).

PCa patients were identified and invited electronically directly through CRN. Patients who used an official digital mailbox (Digipost/eBoks), or the official Norwegian health portal “Helsenorge.no” (since 2020), were invited digitally, while the remaining patients were invited by postal mail. The postal invitations included the questionnaire and a pre-paid return envelope. Patients included from the NoPCR were eligible to participate if they were aged 18 years or older and diagnosed with invasive PCa (n = 8002), regardless of the extent of the disease and treatment choice. PCa patients were excluded if they had other prior cancer(s) or PROM were not assessed within 2 months of diagnosis prior to start of treatment (baseline(T1)) and/or approximately 6 months after treatment (T2) (> 277 days and < 427 from T1 to T2). Simultaneously a reference group from the general population, matched 8:10 by ten-year age groups and geographic region of residence with no previous prostate cancer history, was also invited to participate. The reference group was included to evaluate the longitudinal stability of the EQ-5D-5L in individuals without PCa and to provide a contextual comparator for interpreting changes in the PCa cohort. Although no formal power calculation was performed due to the population-based design, the resulting sample sizes are assumed to provide adequate precision for the planned analyses.

### Demographics and clinical characteristics

Socio-demographic characteristics included age (years), bodyweight (kg), completed education (≤ 12 years) and being married/cohabitant (yes/no) at baseline. For the PCa patients, basic diagnosis- and treatment-related data included time of diagnosis, SEER grade of PCa stages (localized, regional, distant metastases, unknown), and treatment (radical prostatectomy, radiotherapy).

### Patient-reported outcome measures

The PROMs included the disease-specific instruments EORTC-QLQ-C30 and EPIC-26 and the generic instrument EQ-5D-5L, providing a unique opportunity in comparing the generic EQ-5D-5L and disease-specific instruments with respect to validity and responsiveness to change. PROs were assessed at baseline preferably within 2 months of diagnosis (T1) prior to start of treatment, and approximately 6 months after treatment (T2). The PROMs were scored according to published algorithms [17–21].

*The European Quality of Life 5-Dimensions questionnaire (EQ-5D-5L*) is a generic health utility questionnaire consisting of five single items (domains): mobility, self-care, usual activities, pain/discomfort and anxiety/depression (dimensions of health) with ratings on five levels of perceived problems from no problems (1) to being unable to do/having extreme problems [22]. A higher score on EQ-5D-5L reflect more problems. The level scores are presented as global health indices with a weighted total value for health status [21, 23, 24]. The second part is a Visual Analogue Scale (VAS) ranging from 0 (worst health state) to 100 (best health state) and used as an overall measure of perceived health status, Higher scores on EQ-VAS reflect better perceived health. Evidence supports the validity of the EQ-5D-5L for use in the Norwegian general population [18, 19, 21]. In the present study, we used the population norms for UK since the statistical package R did not yet include the updated Norwegian population norms [19].

*The European Organization for Research and Treatment of Cancer Quality of Life Questionnaire (EORTC-QLQ-C30)* focuses on several HRQoL aspects of cancer patients and is not PCa specific [25]. The instrument contains five functional subscales including physical functioning (five items), role functioning (two items), emotional functioning (four items), cognitive functioning (two items) and social functioning (two items), a global health status question, and nine symptom subscales (fatigue, pain, nausea and vomiting, dyspnea, insomnia, appetite loss, constipation, diarrhea and financial impact). A higher score indicates better quality of life for functional scales and global health status, and lower quality of life for symptom scales [25]. Evidence supports the validity of the EORTC QLQ-C30 for use in Norwegian patients with head and neck cancer [26] and the Norwegian general population [27].

*The Expanded Prostate Cancer Index Composite short form (EPIC-26*) is a disease-specific instrument comprises 26 items across the following five domains: urinary incontinence; urinary irritation/obstruction; bowel function; sexual and vitality functions; hormonal disturbance. In addition to measuring functioning within each domain, the instrument also assesses the extent to which patients perceive these areas as problematic, with a recall time the last four weeks [28]. Domain scores were transformed to a 0–100 scale, with higher scores representing better quality of life and functioning [28, 29]. Evidence supports the validity of the EPIC-26 for use in Norwegian non-metastatic PCa patients undergoing radical prostatectomy or prostatic radiotherapy [29].

### Statistical analysis

Descriptive statistics were conducted using IBM SPSS Statistics Version 26.0 software (IBM Corp, Armonk, NY, USA). Paired t-test were performed with latent change scores in a structural equation model (SEM) framework [30], using the R package Lavaan version 6.12 [31]. The SEM models were estimated by using Maximum likelihood.

Change between T1 (pre-treatment) and T2 (post-treatment) was estimated using a latent change score model (LCS), in which a latent variable represents the change for each outcome. This approach was chosen because it allows estimation of correlated change across multiple PROMs within a single multivariate framework and handles missing data efficiently using Full Information Maximum Likelihood (FIML). FIML yields unbiased estimates under missing at random (MAR) assumptions [32], and including outcome variables in the same model provides auxiliary information that supports the plausibility of MAR [33]. Age, SEER stage, and treatment type were not included as auxiliary variables to support the MAR assumption for FIML. Because an LCS model with time points is fully saturated, with zero degrees of freedom and a perfect fit to the data, model fit indices are not informative (df = 0) and no fit indices are reported.

**Fig. 1:**
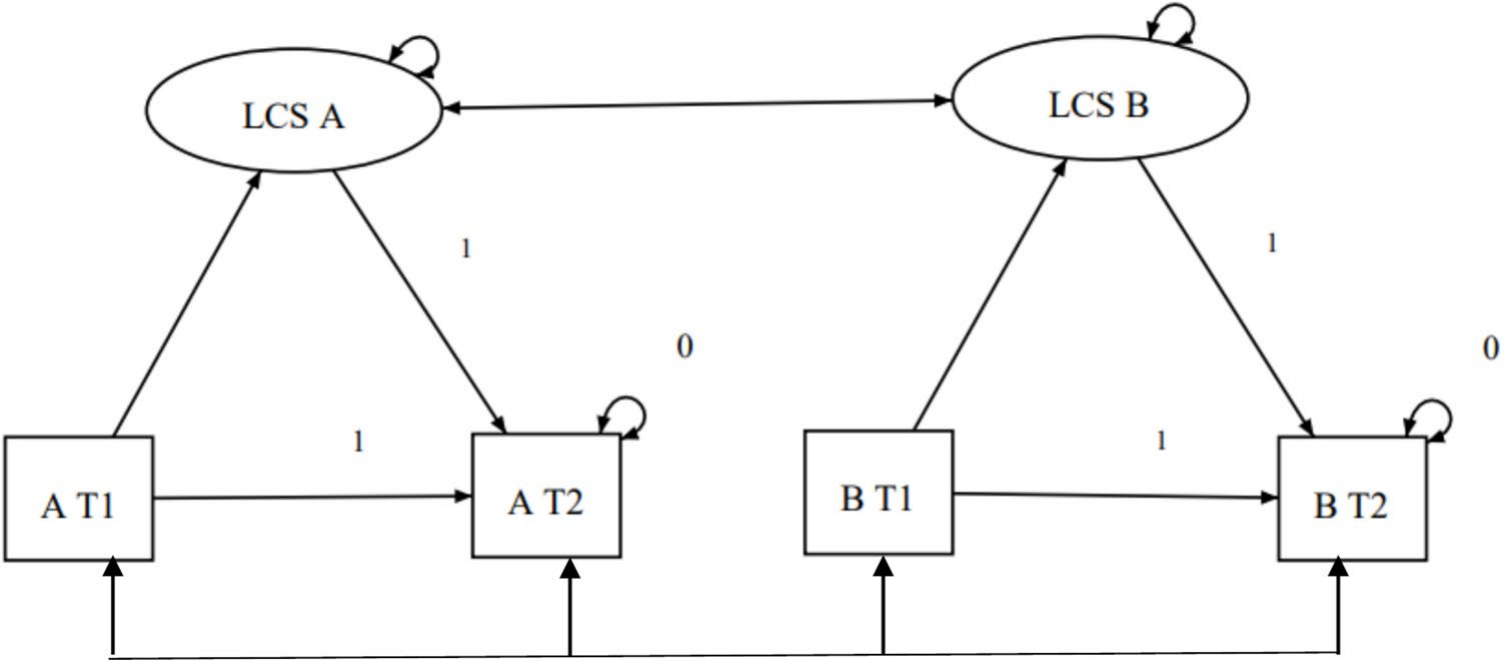
An example of a fully saturated latent change score (LCS) model with two outcome variables (A and B), where T1 is pre-treatment and T2 is post-treatment. Main characteristics of the LCS model: The residual error and intercept of the T₂ variables are fixed at zero, the auto-regressive paths from T₁ to T₂ is constrained to 1, and the paths from the latent change score (LCS) to T₂ is also set to 1

The magnitude of change is expressed by Cohen’s d which was calculated by dividing the change score of the outcome variable by the standard deviation of the baseline score on this variable. A Cohen’s d of 0.20 was considered small effect size, 0.50 as medium effect size and 0.80 as large effect size. Correlations of 0.1, 0.3 and 0.5 were considered as small, medium and large effect sizes, respectively [34]. Figure 1 illustrates the latent change score model structure for two outcome variables (A and B). A visual summary, presented as a bar plot of effect sizes across instruments, was generated using R.

## Ethics

The Regional Committee for Medical and Health Research Ethics South-East approved the establishment of the cohort (REK no. 2015/1294), while the Regional Committee for Medical and Health Research Ethics West approved the present study (REK no. 94540). Study participation was voluntary and based on informed consent, which was provided by participants by returning the questionnaire. Participants could withdraw consent at any time without providing any reason, either online or by contacting the CRN [15].

## Results

A total of 620 participants with PCa (PCa group) and 1776 participants with no previous cancer history (reference group) were included. Demographics of the PCa group and reference group at baseline, as well as clinical characteristics of the PCa group, are given in Table 1. Mean age was 65 years (range 37–79) for the PCa group and 68 years (range 43–96) for the reference group. In total, 75.6% (620 participants with PCa and 1776 referents) answered the questionnaires within 62 days after diagnosis.

**Table 1:** Description of the study population

	**Patients w/PCa** **N = 620**	**Reference group** **N = 1776**	
*Demographic characteristics*			
Age (years), mean (SD)	65.3 (6.52)	68.4 (7.79)	
Lower education, (≤ 12 years)(%)	10.2%	14.4%	
Married/Cohabitant (%)	85.4%	79.6%	
Body weight (kg), mean (SD)	86,6 (12,7)	85,9 (13,9)	
*Clinical characteristics*			
PCa localized (%)	48.5%		
PCa distant metastases (%)	0.2%		
PCa Regional (%)	36.6%		
PCa unknown (%)	14.7 (%)		
*Radiotherapy l*			
Not treated with radiotherapy	39.7%		
Treated with radiotherapy	4%		
Unknown	65.3%		
*Radical prostatectomy* *SEER grade*	91.9%		
Distant	0.2%		
Localized	48.5%		
Regional	36.6%		
Unknown	14.7%		

**Fig. 2:**
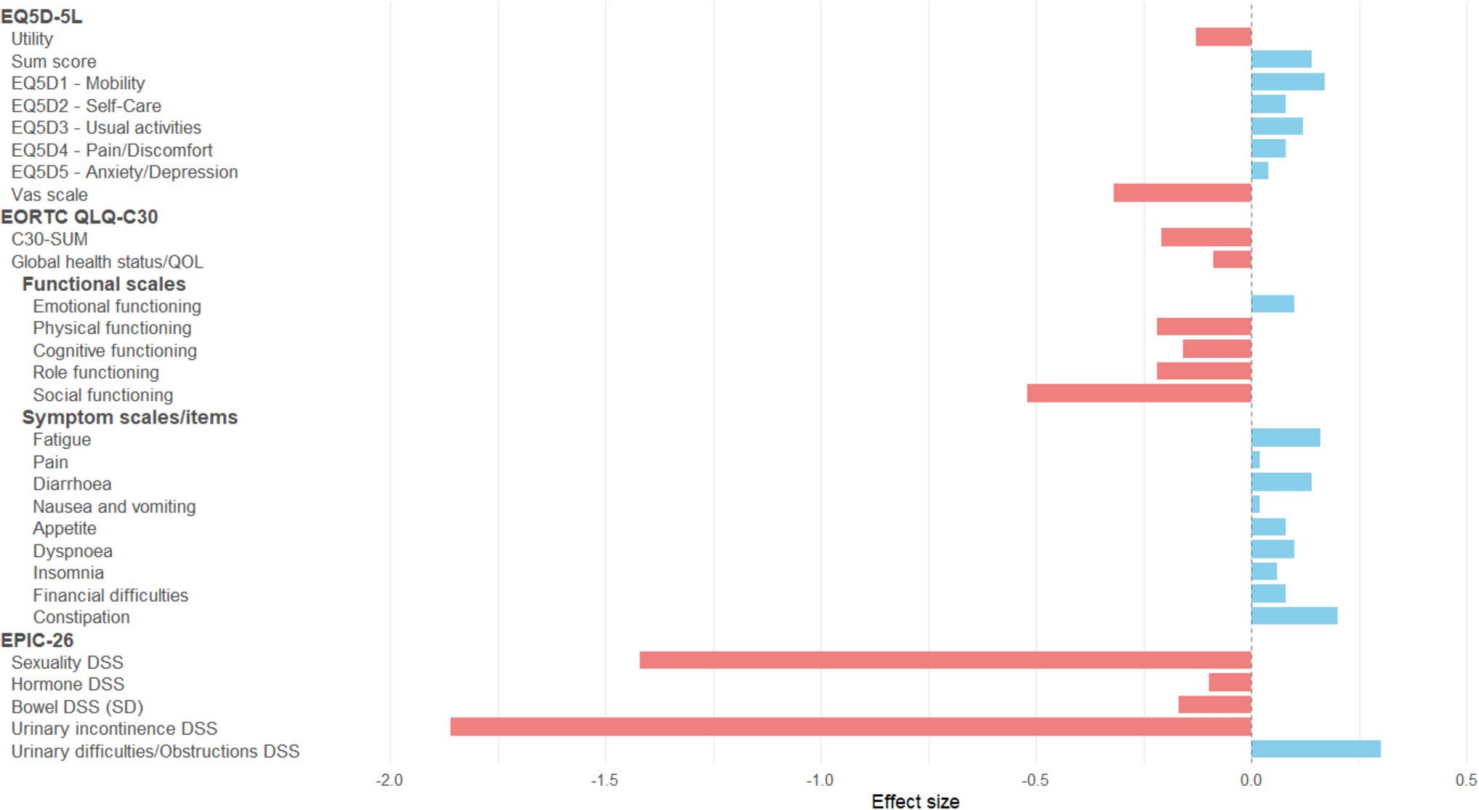
.

An overview of pre- to post-treatment effect sizes across instruments is shown in Fig. 2

Figure 2 Effect sizes (Cohen's d) for pre-to post treatment changes across instruments

### EQ-5D-5L responsiveness

For the PCa group, changes in EQ-5D-5L domain scores from T1 to T2 were statistically significant but small. Three domains (Self-Care, Usual Activities, Pain/Discomfort) increased slightly, suggesting modest deterioration (Cohen’s d = 0.12–0.19), while Mobility showed a slight improvement (Table 2). Changes were below the threshold for small effect sizes. The reference group also showed small deteriorations from T1 to T2 (effect sizes 0.04–0.17), consistent with minor age-related changes.

**Table 2:** EQ5D-5L scores for prostate cancer (PCa) patients (n = 620) before and after treatment compared to the reference group

	**Participants with PCa (N = 620)**					**Reference group (N = 1776)**				
**EQ5D-5L**	**Before** **treatment**	**After treatment**	**Mean** **change**	**P value**	**Cohen’s d**	**Before treatment**	**After treatment**	**Mean change**	**P value**	**Cohen’s d**
Utility	0.93	0.91	-0.02	< 0.001	-0.20	0.92	0.91	-0.01	< 0.001	-0.13
Sum score	6.17	6.49	0.32	< 0.001	0.19	6.29	6.55	0.26	< 0.001	0.14
EQ5D1—Mobility	1.15	1.06	-0.09	< 0.001	0.18	1.23	1.33	0.10	< 0.001	0.17
EQ5D2—Self-Care	1.02	1.04	0.02	< 0.05	0.12	1.05	1.07	0.02	< 0.01	0.08
EQ5D3—Usual activities	1.14	1.23	0.09	< .001	0.19	1.17	1.23	0.06	< 0.001	0.12
EQ5D4—Pain/Discomfort	1.45	1.56	0.11	< .001	0.18	1.60	1.66	0.06	< 0.001	0.08
EQ5D5—Anxiety/Depression	1.41	1.41	0.00	0.91	0.01	1.25	1.27	0.02	< 0.05	0.04
Vas scale	80.39	76.41	-3.98	< 0.001	-0.27	81.11	76.28	-4.83	< 0.001	-0.32

Higher scores on the EQ-5D dimensions and the EQ5D sum score reflect more problems. Whereas higher scores on the EQ-VAS indicate better perceived health. Higher EQ-5D-5L utility index scores also indicate better health

The mean EQ-5D-5L utility index for PCa patients decreased from 0.93 to 0.91 (mean change − 0.02; d =  − 0.20), indicating a small deterioration in overall health status. The reference group showed similarly small declines (0.92 to 0.91; d =  − 0.13) (Table 2).

### EORTC QLQ-C30 responsiveness

For QLQ-C30, most domains showed minor changes (mean change < 5 points). The exception was social functioning, which declined from 88.4 to 78.6 (mean change − 9.85; d =  − 0.52; p < 0.001), indicating a clinically important deterioration (Table 3).

**Table 3:** EORTC-QLQ-C30 scores for prostate cancer (PCa) patients (n = 620) before and after treatment

	**Before** **treatment**	**After treatment**	**Mean change**	**P value**	**Cohen’s d**
**EORTC QLQ-C30**					
C30-SUM	89.22	86.77	-2.45	< 0.001	-0.21
Global health status/QOL	81.80	80.17	-1.63	< 0.05	-0.09
Functional scales					
Emotional functioning	86.84	88.46	1.62	< 0.01	0.10
Physical functioning	93.29	90.51	-2.78	< 0.001	-0.22
Cognitive functioning	90.59	88.33	-2.26	< 0.001	-0.16
Role functioning	89.77	85.32	-4.45	< 0.001	-0.22
Social functioning	88.42	78.58	-9.85	< 0.001	-0.52
Symptom scales/items					
Fatigue	19.56	22.61	3.05	< 0.01	0.16
Pain	13.30	13.68	0.38	0.64	0.02
Diarrhoea	11.02	13.66	2.64	< 0.01	0.14
Nausea and vomiting	2.08	2.24	0.16	0.63	0.02
Appetite	3.53	4.54	1.01	0.10	0.08
Dyspnoea	12.40	14.37	1.96	< 0.05	0.10
Insomnia	19.32	20.83	1.51	0.11	0.06
Financial difficulties	3.85	4.93	1.08	< 0.05	0.08
Constipation	7.81	11.42	3.61	< 0.001	0.20

EORTC-QLQ-C30. Higher scores indicate better quality of life on the functional scales, the global health status scale, and the EORTC QLQ-C30 summary score, but worse quality of life on the symptom scales

### EPIC-26 responsiveness

EPIC-26 showed the largest and clinically most meaningful changes from baseline (T1) to 6 months after treatment (T2). Sexual function declined substantially (65.2 to 27.1; mean change − 38.0; d =  − 1.42; p < 0.001), and urinary incontinence scores declined from 92.7 to 68.4 (mean change − 24.3; d =  − 1.86; p < 0.001), indicating markedly worse urinary control. Urinary irritation/obstruction increased modestly (mean change 4.3; d = 0.30), suggesting more urinary difficulties (Table 4). Changes in the other EPIC-26 domains were minor.

**Table 4:** EPIC-26 scores for prostate cancer (PCa) patients before and after treatment (n = 620)

Patients w/PCa	Before Treatment	After treatment	Mean change	P value	Cohen’s d
**EPIC-26**					
Sexuality DSS	65.24	27.09	-38.14	< 0.001	-1.42
Hormone DSS	89.90	88.66	-1.24	< 0.05	-0.10
Bowel DSS (SD)	93.86	91.91	-1.95	< 0.001	-0.17
Urinary incontinence DSS	92.70	68.37	-24.3	< 0.001	-1.86
Urinary difficulties/Obstructions DSS	84.22	88.54	4.33	< 0.001	0.30

EPIC-26 measuring functioning across five domains, with higher scores representing better functioning (e.g. better urinary control and better sexual function). DSS = domain summary score

### Correlations among change scores

A latent change score model examining associations between changes in EQ-5D-5L and the disease-specific measures is presented in Table 5. Change in social functioning (QLQ-C30) showed small-to-moderate correlations with several EQ-5D-5L domain scores as well as the utility index and VAS (r values up to 0.40). In contrast, correlations between changes in EQ-5D-5L scores and changes in sexual function and urinary incontinence (EPIC-26) were consistently small (r < 0.25), indicating limited overlap between these constructs.

**Table 5:** Correlation between change in EQ5D-5L domains and change in social function (measured by QLQ-C30) and sexual function, urine incontinence (measured by EPIC-26) for Prostate Cancer (PCa) group (n = 620)

EQ5D-5L	Change social Function	Change urine incontinence	Change Sexual function
Change Utility	0.36***	0.24***	0.07
Change EQ5D Sum score	0.40 ***	-0.24	-0.12**
Change Mobility (EQ5D1)	-0.33***	-0.14**	-0.08
Change Self-Care (EQ5D2)	-0.15***	-0.08	0
Change Usual activities (EQ5D3)	-0.31***	-0.19***	-0.12**
Change Pain/Discomfort (EQ5D4)	-0.28***	-0.15***	-0.08
Change Anxiety/Depression (EQ5D5)	-0.13**	-0.16***	-0.02
Change VAS scale	0.35***	0.14**	0.15**

Higher scores on the EQ-5D dimensions and the EQ5D sum score reflect more problems. Whereas higher scores on the EQ-VAS indicate better perceived health. Higher EQ-5D-5L utility index scores also indicate better health. EORTC-QLQ-C30. Higher scores indicate better quality of life on the function scales (social function). EPIC-26 measuring functioning across five domains, with higher scores representing better functioning (e.g. better urinary control and better sexual function)

^*^P-value is significant at the 0.05 level (2-tailed); **P-value is significant at the 0.01 level (2-tailed); *** P < 0.001 is significant at the 0.001 level (2-tailed)

Correlations among the disease-specific instruments (EORTC QLQ-C30 and EPIC-26) were also small across the functional and symptom domains (r ≤ 0.30), as shown in Supplemental Tables 1–2.

### Stratified analyses

Analyses stratified by SEER stage indicated that EQ-5D-5L was more sensitive to differences by disease severity across all domains and the summary score. However, limited power prevented further subgroup analyses.

## Discussion

By using the cancer-specific EPIC-26 and EORTC-C30-QLQ questionnaires, we identified declines in sexual function, urinary incontinence, and social function as important treatment-related domains in subjects with prostate cancer. In contrast, the generic EQ-5D-5L only showed minor changes from pre- to post-treatment in its domains and was not able to clearly discriminate between subjects with and without prostate cancer. Correlations between EQ-5D-5L and the domains in the cancer-specific questionnaire identified to be important for patients with PCa (sexual function, urine incontinence, and social function) indicated low sensitivity to change and therefore, low responsiveness for the EQ-5D-5L domains in this patient group.

The use of a generic measure is often recommended when comparing health status across different conditions [24, 35]. The most attractive features of the generic EQ-5D instrument include its brevity, cognitive simplicity, and widespread translations into many languages. However, the number of levels describing health dimensions are insufficient to detect disease-specific changes (“scale granularity”) [36–38] and important prostate-cancer-related domains, most notably sexual, urinary, and bowel function, are not covered in EQ-5D-5L. In addition, high baseline scores contribute to ceiling effects that further limit its responsiveness.

In the current study, both PCa participants and the reference group reported relatively high EQ-5D-5L scores at baseline, indicating generally good health. Only minor changes were detected in EQ-5D-5L domains from baseline to six months after treatment for the PCa group. In contrast, the cancer-specific PROMs revealed clinically meaningful declines in sexual function (EPIC-26), increased problems with urine incontinence (EPIC-26) and diminished social functioning (EORT QLQ-C30), reinforcing EQ-5D-5L’s limited responsiveness to prostate-cancer-specific health issues. Correlations between EQ-5D-5L change scores and changes in sexual function (≤0.12), urine incontinence (≤-0.19) and social function (≤-0.31) were weak, supporting our hypothesis that EQ-5D-5L does not adequately capture key aspects of post-treatment health change in this population.

Low responsiveness of generic measures in localized PCa aligns with findings from other studies, such as one comparing EPIC-26 with SF-6D and AQoL-6D (Assessment of Quality of Life 6-Dimension), where only the disease-specific instrument demonstrated clinically relevant responsiveness [39]. The magnitude of change observed in our sample was evaluated in relation to recently published minimal important difference (MID) benchmarks from real-world cancer cohorts [40] and prostate-cancer–specific analyses [41]. The reported thresholds suggest that the changes detected by the EQ-5D-5L were small, whereas the disease-specific PROMs captured clinically meaningful deterioration. This may reflect differences between real-world registry data and outcomes derived from controlled clinical trial populations.

Similar concerns have been raised in other disease areas. For example, in a longitudinal study of patients with asthma, the EQ-5D 5L index did not reflect clinically important change in asthma control [42], raising concerns for cost–utility evaluations that heavily rely on Quality-Adjusted Life Year (QALYs), as suggested by the National Institute for Health and Care Excellence guidelines. Conversely, a study designed to detect pre- and postoperative changes in health status of patients after elective foot and ankle surgery found that the EQ-5D-5L was sensitive to such changes [43]. This highlights the importance of confirming the responsiveness of EQ-5D-5L within each disease group and context of use, considering whether the instrument is sufficiently detailed to capture specific functional declines.

We examined validity evidence based on relations to other measures, by assessing correlations between conceptually related constructs measured by different instruments [44]. Because the EQ-5D is a generic instrument, it is not expected to measure the same constructs as a disease-specific instrument. However, some degree of association, typically weak to moderate, can be expected between related constructs. In our study, changes in the EQ-5D-5L domains showed only a weak correlation with changes in sexual function (≤ 0.12) and urine incontinence (≤ 0.19) and social function (≤ 0.31). EQ-5D-5L appeared more sensitive to a higher SEER grade across all domains and in the sum score. Therefore, caution is warranted when using the EQ-5D-5L in an unselected prostate population.

In patients with PCa, the responsiveness of PROMs used will depend on disease status, treatment and prognosis [5–7]. Active treatment of localized PCa often causes adverse side effects tied to urinary, bowel, and sexual dysfunction, domains not directly measured by EQ-5D-5L. This is consistent with prior research showing that generic instruments, including EQ-5D-5L, remain relatively unaffected by treatment in localized PCa [36, 39, 45]. A limited number of studies suggest, however, that EQ-5D-5L may be more responsive to changes in the advanced or metastatic disease stages [46, 47] where skeletal-related events have been shown to impact health status [45]. However, the overall evidence base remains inconclusive, prompting the need for further research [6].

Our sensitivity analyses indicated that the EQ-5D-5L was more responsive for patients with higher SEER stage. However, limited power, particularly only one participant with distant metastases, restricted further analyses. Given the central role of EQ-5D-5L in cost-utility analyses and health technology assessment, limited responsiveness may lead to underestimation of treatment effects and disease burden in PCa populations relying solely on QALYs derived from EQ-5D-5L utilities [36].

Potential ways forward include exploring bolt-on dimensions (e.g., sexual or urinary function) tailored to prostate cancer, developing or validating mapping algorithms from EPIC-26 or QLQ-C30 to EQ-5D utilities, or routinely combining EQ-5D-5L with prostate- or cancer-specific PROMs to ensure comprehensive coverage of relevant health domains. Accordingly, EQ-5D-5L or similarly brief generic instruments should not be used in isolation when assessing health status in PCa populations.

### Strengths and limitations

Strengths of the present study include the population-based design and the availability of diagnostic factors available at the time of diagnosis, though we had limited in-depth information on type of PCa treatment and severity of disease (SEER). The present study is part of a three-year survey on men’s prostate-related health. We have presented and discussed the preliminary and final results with the steering group, including individuals with lived experience in the survey on men’s prostate-related health. As this study has a methodological focus, we have not had continuous involvement of with lived experience in the research process.

Although the response rate is quite reasonable for PROMS in a nationwide registry study, some degree of selection bias cannot be ruled out. Non-responders in large epidemiological studies tend to have more risk factors associated with vulnerable health [48]. On the other hand, it might be that the non-respondents had fewer physical and psychosocial health issues and thus were less motivated to participate in the study than those who participated. Unfortunately, we do not have information on reasons for non-participation, and the nature of any potential bias, if present, remains uncertain.

Our population-based cohort is likely to be more representative of the patient population encountered in clinical practice than those drawn from clinical trials. If selection bias influenced the prevalence of symptoms and functional limitations reported by participants with PCa, it may also have affected estimates of responsiveness to change of the EQ-5D-5L. Assessing these measurement properties was the main focus of our study. In any case, further studies are needed to confirm the performance of EQ-5D-5L when used in other PCa populations.

Finally, we acknowledge that use of the UK EQ-5D-5L value set may introduce some bias in utility estimation due to potential valuation differences between the UK and Norwegian contexts; however, evidence suggests that any impact on the direction or magnitude of the results is likely to be very modest.

Taken together, our findings indicate that although the EQ-5D-5L continues to provide value as a generic instrument, particularly for cross-disease comparisons and health-economic evaluations, its limited coverage of prostate-cancer–specific domains reduce its capacity to reflect the treatment-related changes experienced in this population. In this real-world cohort, clinically meaningful declines were captured only by disease-specific PROMs, underscoring the importance of selecting instruments aligned with the symptom profile of PCa. These considerations are highly relevant when interpreting HRQoL data collected in clinical practice and research settings.

### Conclusion

EQ-5D-5L indicated good health for participants with PCa both before and after treatment, failing to capture clinically relevant problem areas, particularly in terms of sexual function, urine incontinence, which are pertinent for health status. Consequently, the EQ-5D-5L should not be the preferred questionnaire for measuring health status in patients with PCa. If used, it should always be supplemented with a PROM specific for prostate cancer or cancer in general to provide a more complete picture of patient-reported health.

## Supplementary Information

Below is the link to the electronic supplementary material

Supplementary file1 (DOCX 53 KB)

Supplementary file2 (DOCX 16 KB)
