# Supplementary material for: Is EQ-5D-5L sensitive enough to detect treatment-related changes in health status of prostate cancer patients? A nationwide Norwegian longitudinal study from the prostate cancer registry
Source: Qual Life Res. 2026 Feb 4;35(3):64. doi: 10.1007/s11136-026-04178-z (PMC12872650; doi:10.1007/s11136-026-04178-z)
Supplement: Supplementary file 1 — Supplementary file1 (DOCX 53 KB) [file 11136_2026_4178_MOESM1_ESM.docx]

Supplemental Table 1. Correlation between change in EORTC QLQ-C30 - **symptom scales** and change in social function (measured by QLQ-C30) and sexual function and urine incontinence (measured by EPIC-26) and for Prostate Cancer (PCa) group (n=620).

| **EORTC-QLQ-C30**  **- symptom scales** | **Change**  **social function** | **Change**  **urine incontinence** | **Change**  **Sexual function** |
| --- | --- | --- | --- |
| Change fatigue | -0.39*** | -0.16*** | -0.15*** |
| Change nausea and vomiting | -0.20*** | -0.09* | -0.04 |
| Change pain | -0.32*** | -0.12** | -0.05 |
| Change dyspnoea | -0.17*** | -0.07 | -0.08 |
| Change insomnia | -0.24*** | -0.12** | -0.18*** |
| Change appetite loss | -0.23*** | -0.09* | -0.08 |
| Change constipation | -0.24*** | -0.14** | -0.07 |
| Change diarrhoea | -0.06 | -0.04 | 0 |
| Change financial impact | -0.20*** | -0.04 | -0.15*** |
