# Supplementary material for: Is EQ-5D-5L sensitive enough to detect treatment-related changes in health status of prostate cancer patients? A nationwide Norwegian longitudinal study from the prostate cancer registry
Source: Qual Life Res. 2026 Feb 4;35(3):64. doi: 10.1007/s11136-026-04178-z (PMC12872650; doi:10.1007/s11136-026-04178-z)
Supplement: Supplementary file 2 — Supplementary file2 (DOCX 16 KB) [file 11136_2026_4178_MOESM2_ESM.docx]

Supplemental Table 2. Correlation between change in EORTC QLQ-C30 – **function scales** and change in social function (measured by QLQ-C30) and sexual function and urine incontinence (measured by EPIC-26) for Prostate Cancer (PCa) group (n=620).

| **QLQ-C-30**  **- function scales** | **Change social function** | **Change urine incontinence** | **Change Sexual function** |
| --- | --- | --- | --- |
| Change C30 QOL | 0.46*** | 0.22*** | 0.19*** |
| Change C30 Sum score | 0.62*** | 0.24*** | 0.21*** |
| Change Physical functioning | 0.44*** | 0.19*** | 0.19*** |
| Change Social functioning | 1.00 | 0.27*** | 0.25*** |
| Change Role functioning | 0.50*** | 0.24*** | 0.19*** |
| Change Emotional functioning | 0.29*** | 0.14** | 0.09* |
| Change cognitive functioning | 0.18*** | 0.02 | 0.10* |

*P-value is significant at the 0.05 level (2-tailed); **P-value is significant at the 0.01 level (2-tailed); *** P < 0.001 is significant at the 0.001 level (2-tailed).
